# Alternative Treatment in Prostate Pain Syndrome Based on Iranian Traditional Medicine

**DOI:** 10.5812/ircmj.16942

**Published:** 2014-07-05

**Authors:** Seied Amirhossein Latifi, Mohammad Kamalinejad, Bagher Minaiee, Mohsen Bahrami, Shahram Gooran, Alireza Nikbakht Nasrabadi

**Affiliations:** 1 School of Traditional Medicine, Tehran University of Medical Sciences, Tehran, IR Iran; 2 Department of Pharmacognosy, School of Pharmacy, Shahid Beheshti University of Medical Sciences, Tehran, IR Iran; 3 Department of Histology, School of Medicine, Tehran University of Medical Sciences, Tehran, IR Iran; 4 Department of Urology, Sina Hospital, Tehran University of Medical Sciences, Tehran, IR Iran; 5 School of Nursing and Midwifery, Tehran University of Medical Sciences, Tehran, IR Iran

**Keywords:** Prostate, Syndrome, Traditional Medicine

## Abstract

**Introduction:**

Unknown etiology and pathophysiology of prostate pain syndrome (PPS) has led to a lack of proper and competent treatment in modern medicine. According to the guidelines of European Association of Urology (EAU), use of complementary treatments is recommended for PPS. In this preliminary study, analyzing the signs and symptoms of PPS from the viewpoint of Iranian traditional medicine (ITM) was helpful in selecting the appropriate alternative treatment.

**Case Presentation:**

Two male patients diagnosed with PPS were evaluated and treated according to the ITM. Each patient took 15 mL oxymel 45 minutes after lunch and dinner. For each patient, four clinical visits were made with one week intervals and the validated Farsi version of international prostate symptom score (IPSS) and numeric pain rating score (NPRS) were completed for them.

**Conclusions:**

Considering the fact that other major pathological causes are ruled out, many of the symptoms and signs observed in these patients were similar to those associated with flatulency-related diseases in ITM. Selecting treatment with oxymel was based on this view and led to improvements in the digestive and urinary symptoms according to Farsi version of the IPSS and NPRS.

## 1. Introduction

According to the European Association of Urology (EAU) guidelines on chronic pelvic pain, prostate pain syndrome (PPS) is defined as “persistent or recurrent episodic prostate pain associated with symptoms suggestive of urinary tract and/or sexual dysfunction with no proven infection or other obvious pathology" (1). In spite of several multifactorial hypotheses, the etiology of PPS remains unknown. According to the EAU guidelines, specific diseases with similar symptoms, i.e. confusable diseases, should be excluded and a validated symptom and quality of life scoring instrument should be applied to assess PPS. Additionally, utilizing complementary treatment within a framework of multimodal treatment options is recommended for PPS (1 2 3). This study aimed to report the results of alternative diagnostic and therapeutic approaches to PPS based on Iranian traditional medicine (ITM) concepts. Two male patients presented with a final diagnosis of PPS in the Urology Clinic of Sina Hospital were evaluated and based on the obtained results, Sikanjabin (oxymel) was prescribed. Treatment of these two patients with oxymel improved the PPS symptoms.

## 2. Case Presentation

Two male patients inhabited Tehran, Iran, were referred by their urologist from the Urology Clinic of Sina Hospital, Tehran University of Medical Sciences, to Iranian Traditional Medicine Clinic for further evaluation based on Avicenna’s viewpoint. A summary of their characteristics is presented in Table 1.

The main complaint of patient 1 was periodic and sharp pain in the perineal and suprapubic regions. Symptoms were aggravated after drinking cold water and flatulent foodstuffs. He also complained of increased urinary frequency and dysuria following urination. He experienced hesitation at initiation of urination with slow stream and straining. In recent years, his level of physical activity decreased and became mostly sedentary after the patient changed his job to become a tailor, which had further aggravated his symptoms. A sonography of the scrotum had normal findings. Prescribed medications for chronic prostatitis included long-term cycles of antibiotics, despite no evidence of bacterial infection in urine cultures or prostatic secretions, an alpha-blocker, and an analgesic. He experienced no relief with any of these oral medications. The patient reported cholecystectomy because of gallbladder stone about four years ago. He complained of bloating and distention, heartburn pain, and discomfort in epigastrium and used omeprazole for his duodenal ulcers.

**Table 1.** Patients Characteristic^a^

Characteristic	Patient 1	Patient 2
**Demographic Data**		
Age, y	46	40
Job	Tailor	Driver
Education	Low	Low
BMI, kg/cm^2^	25-29.9	25-29.9
Smoking	Yes	No
Parity	Yes	Yes
**Duration of Symptom, y**	> 2	> 1
**Previous treatment**	Tamsulosin, ofloxacin, and baclofen	Tamsulosin, ofloxacin, baclofen, nitrofurantoin, and fluoxetine
**Diagnostic testing**	Negative	Negative
**Comorbid Conditions**	Low back pain and duodenal ulcer	Irritable bowel syndrome and chronic sinusitis
**Symptoms**	Perineal and suprapubic pain, dysuria, frequency, hesitancy	Postmicturition pain, dysuria, frequency, urinary retention, hesitancy

^a^ Abbreviation: BMI, body mass index.

Patient 2 was referred with chief complaints of postmicturition pain in the tip of the penis and testis with dysuria. He described a feeling of chest tightness and nausea as well as urinary retention. He had a history of an episode of urinary tract infection in the preceding years without any recurrence. He reported increased urinary frequency and a burning sensation in his urethra that had caused a hesitation to initiate. Sonography of the scrotum, kidney, and bladder revealed normal structures and a moderate postvoid residual volume. He was a driver and was in sitting position during most of his day at work. Similar to patient 1, this patient had repeated long-duration series of antibiotics, alpha-blocker, and analgesic despite no bacterial infection of prostate secretions or urine on the laboratory reports. He also reported heartburn, bloating and distention with feeling of fullness in throat, and gastroesophageal reflux. Other complaints included a feeling of incomplete bowel movements and pain in the anus and rectum.

Physical examination of the abdomen, genitourinary tract, and a digital rectal examination (DRE) to assess rectal soft tissue were performed for both patients. There were no clinically significant findings except moderate suprapubic tenderness and some tender points in the groins, proximal thighs, and perinea. No tumoral lesion or glomerulation was observed in the cystoscopy of the patients. Bladder biopsy and hydrodistention were also performed. Bladder biopsy and urine cytology results were negative for malignancy. Moderate prostate enlargements in the sonography could not explain the observed clinical symptoms in patients.

### 2.1. Intervention

The validated Farsi version of the international prostate symptom score (IPSS) (4) and numeric pain rating score (NPRS) were used for both patients as a pre-treatment symptom quantifier and posttreatment monitoring tool. Internal consistency for the Farsi version of IPSS was 0.7 by Cronbach’s α test (4). Each patient took 15 mL oxymel 45 minutes after lunch and dinner. Reports of their conditions were prepared every seven days for four weeks. The results are displayed as pre-treatment score and 30^th^ day of treatment score (post-treatment score) in Table 2. Although the formulation of oxymel is described in details in the British Pharmacopoeia (1898), the German Pharmacopoeia (1872), and the French Codex (1898), we prepared it according to Avicenna's formulation in his Canon of Medicine (5, 6, 7). Most sources have described the formulation of oxymel as four spoons of sugar plus one spoon of vinegar.

**Table 2.** Pre-treatment and Post-treatment Symptom and Quality of Life Impact Score^a^

Measure	Patient 1		Patient 2	
	Pre-treatment	Post-treatment	Pre-treatment	Post-treatment
**Farsi Version of IPSS, (41)**	34	11	31	10
**NPRS, (0-10 scale)**	8	3	6	2
**Total Score, (51)**	42	14	37	12

^a^ Abbreviations: IPSS, international prostate symptom score; and NPRS, numeric pain rating scale.

## 3. Discussion

The differential diagnosis of bladder pain and urinary symptoms have been discussed in details in Avicenna's Canon of Medicine Book III, Part 19, in two sections (8):

In section 1, Bladder-related diseases, pain is acknowledged as the most common symptoms of bladder disease. Differential diagnosis of bladder pain have been discussed in details in this section. Bladder paresis, weakness, and hematoma are also discussed in this section. 

In section 2, Urination-related diseases, Avicenna classifies urinary symptoms and differentiates storage symptom from voiding symptom; in addition, he offers definitions and accounts of the causes and different diagnostic methods for dysuria, incontinence, difficult urination, polyuria, intermittency, oliguria, anuria, enuresis, diabetes, and hematuria.

Common causes of lower urinary tract symptoms based on Avicenna's system of concepts are:

1. bladder calculus;
2. bladder/urethra ulcers and pustules;
3. inflammation, inflation, tumor, and mass;
4. obstructive and anatomic causes (scars, wart, hematoma, pus, and abnormality);
5. neurologic causes (sensory and motor dysfunction of bladder or sphincter);
6. trauma (bladder, urethra, and cerebrospinal);
7. causes related to involved organs (liver, kidney, genital tract, and gastrointestinal tract) through digestive system;
8. urine causes; and
9. wind or gas ("Reeh" in Arabic).

Nowadays almost all of the aforementioned causes of lower urinary tract symptom are detected through clinical and paraclinical procedures with high sensitivity and accuracy except for the last mentioned cause. Based on Avicenna's system, these symptoms are caused by flatulency in pelvic organs. In the context of traditional medicine, "Reeh" is a current flow with blasting force in the body that acts like the “wind” in the nature and plays a crucial role in the pelvic organs ducts to facilitate execratory functions such as urination, defecation, and erection-ejaculation in physiologic conditions. Therefore, we can consider it as an equivalent to the peristaltic waves in excretory organs such as bowel, ureter, and the bladder. Any disturbance in the production process of "Reeh", either in its quantity or quality, leads to not only failure of these physiologic functions but also flatulence and spasms that produce pains with specific characteristics. This pain is reported to be distending, stabbing, or onerous in quality. It is a nonlocalized shifting pain with a sudden and sever onset that can last short at certain times, especially in good digestive conditions. This specific clinical symptomatic diagnosis distinguishes traditional medicine diagnostic approach from modern approaches. Therefore, chronic pelvic pain and lower urinary tract symptoms that have unknown etiology and pathophysiology can be considered in this group. Due to the identical physiologic performance of "Reeh" in the excretory organs such as the gastrointestinal and urogenital tracts, it is reasonable to associate PPS with multiple organs in these patients in the absence of reliable pathologic evidences. Evaluation of the two patients with final diagnosis of PPS in the framework of traditional medicine reveal that poor gastrointestinal digestive function disturbs production of a potent" Reeh" and finally, results in the impairment of the excretory function of related organs and lead to flatulency. Avicenna emphasizes that pains in the bladder and kidney that are due to this cause can be reduced by fasting and proper digestion (8). Following the approach of ITM, oxymel was used for these patients to improve food digestion because of its antiflatulent and diuretic effects. Considering the fact that oxymel has been used as an ancient healing drink over centuries, since the time of Hippocrates up to modern times, for treatment of various diseases such as persistent coughs, fevers, jaundice, and dehydration (5, 7), new evidence-based investigations for determining the therapeutic effects of this low-price natural product, especially in chronic pelvic pain syndromes, is necessary. An improve in the digestive and urinary symptoms, decrease in pelvic pains, and an enhanced quality of life in both patients showed that alternative treatments could be effective when chosen based on correct assumptions for syndromes with unknown causes in modern medicine. For these two patients, modification of lifestyle, manual therapy including massage, and cupping were also prescribed in order to attain final treatment and preventing relapse of syndromes. The results of these measures can be examined and analyzed in future studies.
